# Functional MRI during Hippocampal Deep Brain Stimulation in the Healthy Rat Brain

**DOI:** 10.1371/journal.pone.0133245

**Published:** 2015-07-20

**Authors:** Nathalie Van Den Berge, Christian Vanhove, Benedicte Descamps, Ine Dauwe, Pieter van Mierlo, Kristl Vonck, Vincent Keereman, Robrecht Raedt, Paul Boon, Roel Van Holen

**Affiliations:** 1 Medical Image and Signal Processing Group, Ghent University-iMinds Medical IT department, Ghent, Belgium; 2 Laboratory for Clinical and Experimental Neurophysiology, Neurobiology and Neuropsychology, Ghent University Hospital, Ghent, Belgium; University Medical Center Goettingen, GERMANY

## Abstract

Deep Brain Stimulation (DBS) is a promising treatment for neurological and psychiatric disorders. The mechanism of action and the effects of electrical fields administered to the brain by means of an electrode remain to be elucidated. The effects of DBS have been investigated primarily by electrophysiological and neurochemical studies, which lack the ability to investigate DBS-related responses on a whole-brain scale. Visualization of whole-brain effects of DBS requires functional imaging techniques such as functional Magnetic Resonance Imaging (fMRI), which reflects changes in blood oxygen level dependent (BOLD) responses throughout the entire brain volume. In order to visualize BOLD responses induced by DBS, we have developed an MRI-compatible electrode and an acquisition protocol to perform DBS during BOLD fMRI. In this study, we investigate whether DBS during fMRI is valuable to study local and whole-brain effects of hippocampal DBS and to investigate the changes induced by different stimulation intensities. Seven rats were stereotactically implanted with a custom-made MRI-compatible DBS-electrode in the right hippocampus. High frequency Poisson distributed stimulation was applied using a block-design paradigm. Data were processed by means of Independent Component Analysis. Clusters were considered significant when p-values were <0.05 after correction for multiple comparisons. Our data indicate that real-time hippocampal DBS evokes a bilateral BOLD response in hippocampal and other mesolimbic structures, depending on the applied stimulation intensity. We conclude that simultaneous DBS and fMRI can be used to detect local and whole-brain responses to circuit activation with different stimulation intensities, making this technique potentially powerful for exploration of cerebral changes in response to DBS for both preclinical and clinical DBS.

## Introduction

Deep brain stimulation (DBS) is a widely accepted treatment for advanced Parkinson’s disease (PD) [1,2] and is a promising therapy for other neurological and psychiatric disorders, such refractory epilepsy [3–5]. DBS is an adjustable and reversible functional therapy during which electrical pulses are delivered to specific brain targets by means of stereotactically implanted deep brain electrodes. Despite its remarkable clinical success, the precise mechanism of action remains to be elucidated.

A better understanding of the neuronal networks modulated by DBS locally and on a whole-brain scale, is therefore required to improve treatment efficacy. Most studies, namely electrophysiological and cellular studies, have investigated the local effects of DBS. However, visualization of whole-brain effects of DBS requires functional imaging techniques, such as functional Magnetic Resonance Imaging (fMRI), Positron Emission Tomography (PET) and Single Photon Emission Computed Tomography (SPECT), that allow investigating whole-brain effects of locally delivered electrical current pulses. With this study our aim is to investigate DBS-induced global neuronal network activation in healthy rats by means of fMRI.

FMRI is an important non-invasive technique to investigate functional processes in the brain and is mainly used to image brain activation. It uses blood oxygenation level-dependent (BOLD) contrasts, which are related to local field potentials (LFP) or postsynaptic activity [6–7]. A stimulus-induced increase of neuronal, and thus electrical, activity causes an increase in the consumption of oxygen, resulting in fluctuations in the levels of paramagnetic deoxyhemoglobin. The changing level of cerebral deoxyhemoglobin, as response to a stimulus, can be measured using a magnetic field, and it is referred to as the BOLD response to the stimulus. A major advantage of fMRI is the possibility to examine a whole-brain response, as opposed to electrophysiological recordings and neurochemical studies that are characterized by restricted spatial sampling. Additionally, fMRI has a superior spatial and temporal resolution compared to PET and SPECT, and fMRI allows the investigation of the modulatory potential of electricity on a subcortical-cortical pathway, because stimulation parameters can easily be varied during a single fMRI scanning session [7–10]. Several PET or SPECT scanning sessions would be needed to investigate the neuromodulatory effects of DBS, as it is a single PET or SPECT scanning session does not allow the acquisition of the physiological changes induced by making selective and reversible changes in the stimulation parameters during a single PET or SPECT scanning session. Lastly, fMRI does not require the use of a radioactive tracer.

Recent studies have shown that simultaneous DBS fMRI can be used for the study of in vivo network activation on a large spatial scale [11–14]. These studies aimed to identify the global effect of subthalamic nucleus (STN) or ventral posterior thalamic (VP) DBS in rodents or non-human primates. In general, these studies demonstrate that STN DBS or VP DBS induces a positive BOLD response within several brain structures of the motor/sensory network and the basal ganglia, indicating thalamic-cortical connectivity and the potential of DBS fMRI studies to investigate the function of a particular neuroanatomical pathway.

In this study, stimulation of the hippocampus was chosen because there is considerable evidence that the hippocampal formation is involved in seizure initiation in Temporal Lobe Epilepsy (TLE), the most common type of epilepsy [15], and it has the lowest seizure threshold of all brain structures [16–18]. Velasco et al. discovered that unilateral hippocampal DBS (hDBS) decreased interictal and ictal epileptiform activity in refractory TLE patients [19–21]. These findings were confirmed in other clinical trials [22–25] and in animal experimental studies [26–28].

The hippocampal formation includes the dentate gyrus, CA1/2/3 and the subiculum, and the perforant pathway provides a fairly well conserved connection from the entorhinal cortex to all hippocampal substructures [29]. The rat hippocampus is anatomically and physiologically well defined and has recently been investigated with fMRI [30–35]. In general, these studies show that electrical stimulation of the rat hippocampus primarily excites the hippocampal formation and observe secondary fMRI responses in target regions of the hippocampus, such as the septum, striatum, nucleus accumbens (NAcc)/medial forebrain (mFB), substantia nigra, anterior cingulate cortex (ACC) and sensory cortex (SC). These findings indicate that stimulation of the hippocampal formation can activate the mesolimbic pathway [29–34]. However, these studies are limited to acute stimulation and do not use a chronically implanted DBS-electrode, meaning their experimental protocol cannot be used for longitudinal studies. Other DBS fMRI studies have used chronically implanted electrodes where they targeted the thalamic structures and could reveal thalamic-cortical connectivity [36–37]. Besides longitudinal investigation of DBS, a chronically implanted electrode allows for a post-surgical recovery to minimize acute tissue inflammation and for electrode fixation with dental cement to minimize electrode movement during acquisition. To our knowledge this is the first DBS fMRI study with a chronically implanted MRI-compatible electrode for hippocampal DBS. In addition, we chose Poisson distributed stimulation over regular distributed high frequency stimulation because irregularity in interpulse intervals seems to induce more potent anti-seizure effects [28,38]. It has also been suggested that Poisson distributed DBS may cause disruption of the generation and propagation of synchronous epileptic activity [39]. We also decided to investigate the effect of DBS in the healthy brain in order to rule out the diseased brain as a confounder.

Proper anesthetic use is crucial for successful functional imaging in rodents. It is known that the use of medetomidine, a selective alpha-2-adrenoreceptor agonist, as an anesthetic agent results in a reliable stimulus-induced BOLD contrast. Additionally, it can be used in longitudinal studies, since it is administered subcutaneously requiring no catheterization, and the animal is maintained in a free breathing state requiring no intubation [12,40–42]. Another major benefit of the alpha-2-agonist is that its effect can be rapidly reversed by administration of alpha-2-antagonists and that it has a short in vivo half-life, which also favors the use of medetomidine to be used in chronic follow-up studies [40]. Using the suggested dose of medetomidine, the animal is under sedation rather than full anesthesia, and a reliable BOLD signal can be detected.

With this research, our aim is to investigate the whole-brain effect of therapeutic high frequency DBS in healthy rats and to characterize the responses to different stimulation intensities by means of fMRI. This is significant because despite the remarkable clinical success of DBS, there are still non-responders to the treatment. Therefore a better understanding of how hippocampal DBS and its stimulation parameters modulates neural circuitry is necessary to improve treatment efficacy in patients. Successful translation of this research to patients might reduce the number of non-responders to this expensive and invasive treatment.

## Materials and Methods

### Animals

Seven adult male Sprague-Dawley rats (200–250g body weight; Janvier, France) were used for the fMRI experiments. All rats were treated according to guidelines approved by the European Ethics Committee (decree 2010/63/EU). All experimental procedures were approved by the Animal Experimental Ethical Committee of Ghent University Hospital (ECD 13/14). The animals were kept under environmentally controlled conditions (12h normal light/dark cycles, 20–23°C and 40–60% relative humidity) with food and water ad libitum.

### Surgery

**Fig 1:**
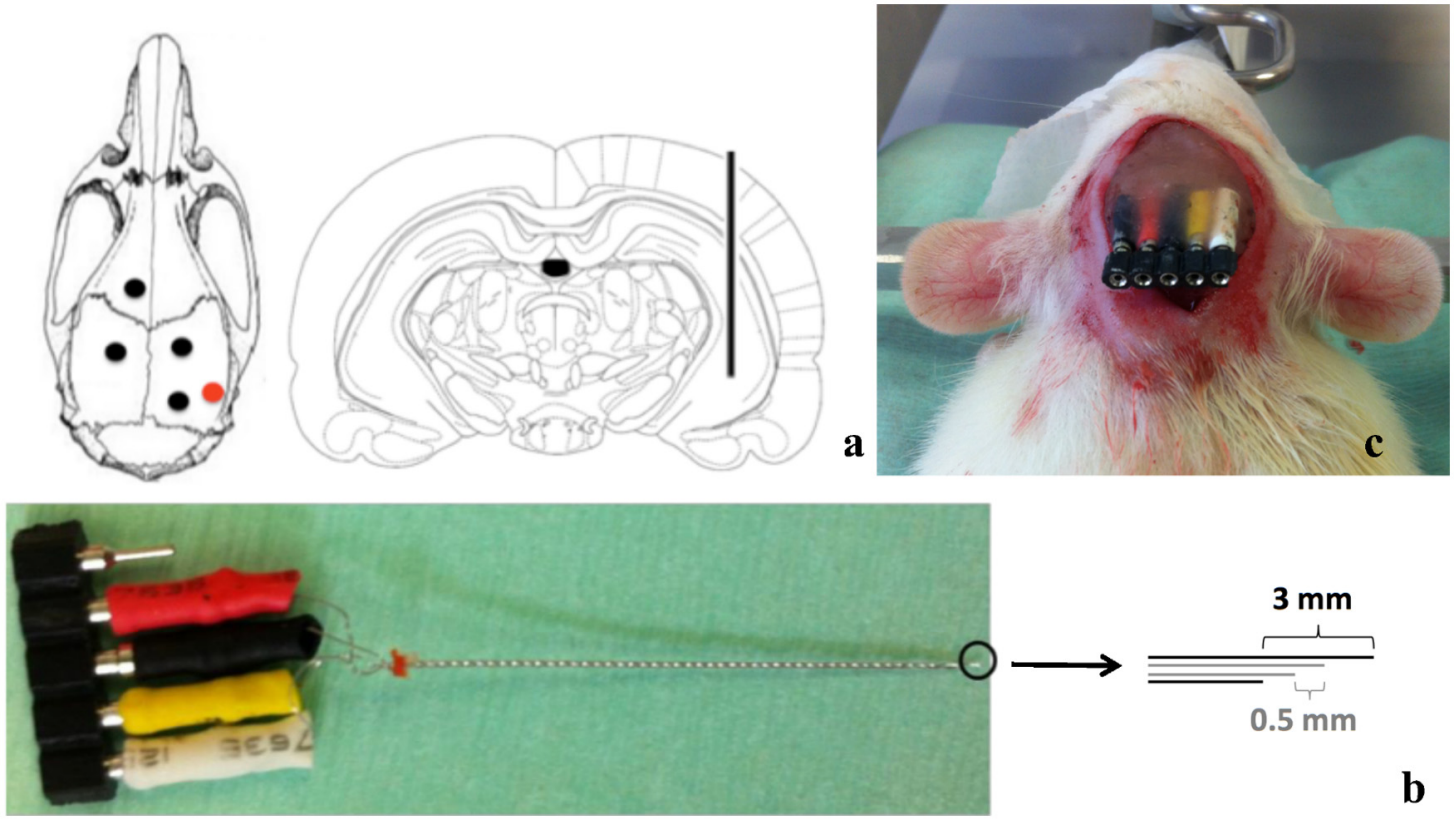
DBS-electrode specifications. (a) The quadri-polar DBS-electrode was stereotactically implanted in the right hippocampus (AP -5.6, DV -7.4, ML +5.2 relative to Bregma [43]) together with four anchor screws. The outer two electrodes are used for stimulation and the inner two electrodes for iEEG. The red dot represents the electrode insertion location on the skull. The black dots indicate anchor screw positions. (b) Illustration of the quadri-polar DBS-electrode. The MRI-compatible electrode is custom made by twisting together four platina iridium wires. The unused connection pin is reserved for connection to a ground-electrode, which is subcutaneously implanted in the neck. (c) Illustration of the DBS-electrode after fixation to the skull.

The rats were anesthetized with a mixture of isoflurane (5% for induction, 2% for maintenance) and medical O_2_. After exposure of the skull, five small burr holes were drilled: four for the positioning of nylon anchor screws (Bilaney consultants GmbH, Germany) and one for the insertion of the quadri-polar DBS-electrode. This DBS-electrode was inserted stereotactically in the right hippocampus (AP -5.6mm, ML +5.2mm, DV -7.4mm relative to bregma) [43]. Each DBS-electrode was custom-made by twisting together four PFA-coated Platinum Iridium wires (A-M Systems, WA, U.S.A.), with 140μm diameter. The most ventral and dorsal electrode contacts were used for stimulation purposes. The distance between the tips was 3mm. These two outer electrodes of the quadri-polar probe were different poles of the same stimulating electrode, providing a bipolar stimulation. The design of the electrode is based on previous studies of DBS in rat models for temporal lobe epilepsy, where bipolar DBS with a 3mm gap between poles was shown to be therapeutically effective [28,38,44]. The two inner wires were used for intracranial electro-encephalographic (iEEG) recording. The distance between these two tips was 0.5mm. The electrode and its placement are shown in Fig 1.

For impedance optimization, all electrodes were electrolytically cleaned. The impedance was checked immediately before implantation and did not exceed 70kOhms (IMP-2, Bak electronics, Sanford Florida, USA). The stimulator (DS4 Bi-phasic Stimulus Isolator, Digitimer Ltd, Hertfordshire, England) can generate up to 48 V, meaning the impedance cannot be over approximately 74kOhms for a stimulation intensity of 650μA (i.e. the maximal administered intensity during threshold determination). One additional polyimide coated stainless steel wire (Bilaney, Germany), with 125μm diameter, was inserted subcutaneously as a ground. The DBS-electrode and the ground-electrode were attached to a connector that was fixed to the screws and the skull with acrylic dental cement. Rats were allowed seven days of post-surgical recovery, during which they were handled.

### Deep Brain Stimulation

All rats were subjected to the same DBS paradigm, namely a bipolar Poisson distributed unilateral hippocampal stimulation. The stimulation paradigm was a five-minute-block-design paradigm with five cycles consisting of 40 seconds of rest (stimulation OFF) followed by 20 seconds of stimulation ON. The stimulation itself consisted of a series of biphasic, charge-balanced square-wave pulses with a pulse width of 100μs. The mean frequency of the stimulation paradigm was 130Hz and the inter-stimulus intervals were Poisson distributed with a mean and variance of 1/130s. Pulses were delivered to the outer two electrode contacts of the quadri-polar MR-compatible DBS-electrode using a constant current linear isolated stimulator, which was triggered by a data acquisition card (NI-DAQ USB-6343, National Instruments, Austin, TX, U.S.A.) that was connected to a standard PC. For each rat the DBS paradigm was applied ten times using five different stimulation intensities, which were determined individually based on the subject’s seizure threshold. This threshold was obtained one day after the postsurgical recovery period. The five different stimulation intensities were set at 10%, 30%, 50%, 70% and 90% of the minimal stimulus intensity that gives rise to an epileptic seizure. This seizure threshold was determined by applying 15s of stimulation, with parameters identical to those applied during the fMRI acquisitions, followed by 60s of rest, during which the iEEG was evaluated for epileptic discharges. For every stimulation episode, stimulation intensity was augmented with 25μA until an ictal discharge was observed on the iEEG. All rats were sedated with medetomidine during this process and the thresholding itself started after at least one hour of continuous infusion of medetomidine. The detection of ictal discharges was performed by recording the iEEG-signal using the inner two wires of the quadri-polar DBS-electrode. The iEEG-signal was amplified using an MRI-compatible amplifier (Brain Products, Gilching, Germany). Ictal discharges were characterized on the iEEG by spiking activity with an amplitude exceeding at least three times the baseline amplitude and with a frequency exceeding 5Hz for at least 10 seconds [45–47]. The seizure thresholds for the 7 rats were: 275, 400, 550, 550, 575, 600 and 650μA; resulting in a mean threshold of 515μA, with a standard deviation of 130μA. The variation of seizure threshold between rats might be related to the intrinsic biological variability between rats, or to differences in electrode placement, since more or less fibers are reached depending on the placement. However, since the distance between stimulation poles of our electrodes is rather large (3mm), variations in electrode placement might have less of an impact, compared to commercial electrodes where there is no, or a very small, distance between the tips. The variation in seizure threshold cannot be related to the impedance because the stimulator corrects for this internally. The DS4 stimulator does this by modifying the voltage to obtain a specific current, and can generate up to 48V.

Because the stimulation intensity causing the ictal discharge was subject-dependent, the length of stimulation is dependent on the subject in during the thresholding step, preceding the actual fMRI experiment. During fMRI the length of stimulation was the same for all subjects. The stimulation paradigm was a five-minute-block-design paradigm with five cycles consisting of 40 seconds of rest (stimulation OFF) followed by 20 seconds of stimulation ON. This paradigm was repeated ten times (using five different stimulation intensities) within one fMRI scanday. Thus the total length of time in which the rats were stimulated was 10 minutes for each scanday. Every subject underwent fMRI on three different scandays, meaning that the DBS paradigm was applied thirty times (i.e. 30 minutes) in total. No stimulation was applied post fMRI.

### Functional MRI

**Fig 2:**
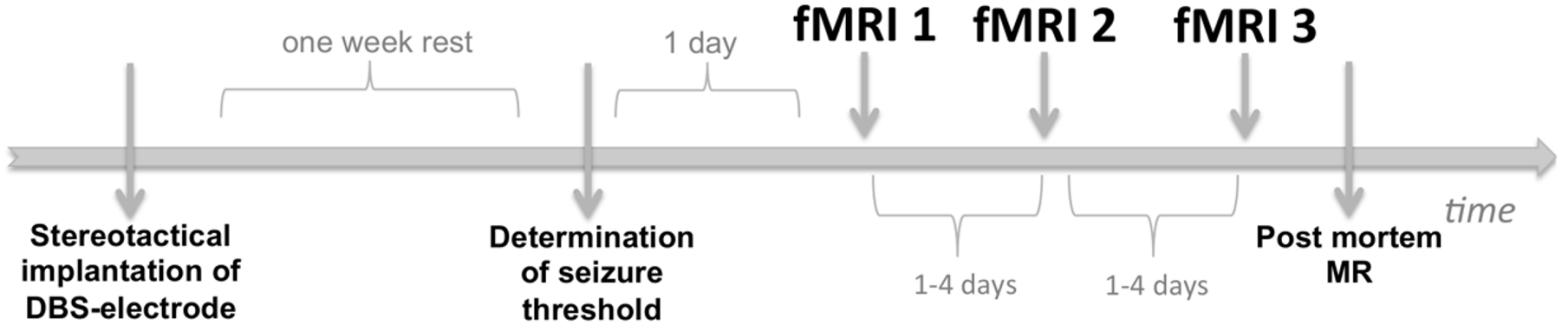
Timeline of the experimental imaging protocol. The animals were allowed one week of post-surgical recovery, after which the seizure threshold was determined. One day after threshold, the 1st series of fMRI datasets were acquired. The 2nd and 3rd series of fMRI datasets were acquired 1 to 4 days after the first set was acquired, depending on scanner availability. Finally rats were sacrificed, the DBS-electrode was removed with high caution and an MR-scan was acquired.

The timeline of the experimental protocol is shown in Fig 2. Every subject underwent MR-acquisitions on three different timepoints. The first timepoint was one day after the threshold determination for all animals. Depending on scanner availability, the time between scanning days ranged from one day up to four days. Electrode impedance was checked at the start of each scanning day, by connecting an impedance meter (IMP-2, Bak electronics, Sanford Florida, USA) to the stimulation poles of the DBS-electrode using 1kHz sine wave testing stimuli. The currents used within the set-up are of magnitude < 30nA, meaning this set-up can be used in vivo. The impedance did not exceed 70kOhms in 4/7 subjects, and did not exceed 80kOhms in 3/7 subjects, which is still ok to administer up to 90% of the highest seizure threshold.

All acquisitions were performed under medetomidine anesthesia. All rats were initially anesthetized with a mixture of isoflurane (5% for induction and 2% for maintenance) and medical O_2_. A bolus of 0.05mg/kg medetomidine was injected subcutaneously, and isoflurane was stopped 10 minutes afterwards. Continuous subcutaneous infusion of medetomidine (0.1mg/kg/hour) was started 15 minutes after the bolus injection to maintain sedation. At least 30 minutes was allowed for equilibration before the actual fMRI acquisition was started. After 2 hours the infusion rate was increased to 0.3 mg/kg/hour. After stepping the infusion rate, a 30 minutes equilibration period was allowed before continuing fMRI acquisitions. Previous fMRI studxies have shown that tripling the infusion rate after 2.5 hours resulted in a prolonged period of similar sedation level up to 6 hours, which could not be achieved with a constant infusion rate [42]. After the acquisition atipamezole (0.03 mg/kg) was administered to reverse the effects of the anesthesia.

MR images were acquired on a 7T system (Pharmascan 70/16, Bruker, Ettlingen, Germany) using a rat head volume coil in order to optimally acquire BOLD responses in deeper subcortical brain structures, such as the hippocampus. The head of the animal was carefully secured in the Bruker rat restrainer using a tooth bar and two flexible cushions on the two sides of the head, before placement inside the magnet. Rat body temperature was maintained at ~37°C by a water-circulated heating pad. Magnetic field homogeneity was optimized in two steps. First- and second-order shims were applied on the global volume, followed by local first-order shims on a volume of 4 x 6 x 12mm^3^ using MAPSHIM. BOLD fMRI acquisitions were performed using a multi-slice single-shot gradient echo echo-planar imaging sequence (GE-EPI). Twelve interleaved slices were acquired with TE = 20ms, slice thickness = 1 mm, matrix size = 80 x 80, FOV = 2.5 x 2.5cm^2^ and voxel size = 0.312x0.312x1 mm^3^. Each fMRI run consisted of 150 repetitions with TR = 2s (total 5 min), corresponding to the duration of the stimulation protocol. Ten GE-EPI runs (i.e. two times five stimulation intensities in randomized order) were acquired with a 5 min rest period between scans for neurovascular recovery and to minimize bias from potential DBS induced heating and tissue damage. For anatomical reference, T2 weighted anatomical images were obtained using a Turbo RARE sequence with TR = 6345ms, TE = 37ms, slice thickness = 0.6mm, matrix size = 276 x 320, FOV = 3 x 3,5cm^2^.

After completion of the experiment the animals were euthanized with an overdose of pentobarbital (150mg/kg, i.p.), and the electrode was removed cautiously. The electrode was inspected under the microscope to assess whether any tissue was removed together with the electrode, which was in our study never the case. Thereafter, a post-mortem structural MR was acquired for electrode path verification purposes and for potential tissue damage evaluation. T2 weighted anatomical images were obtained with a spatial resolution of 100μm x 100μm in the axial plane, i.e. smaller than the thickness of the electrode and thus the lesion. We made sure the positioning of one slice was at the level of the electrode. We believe this method is sufficiently accurate in assessing the correct electrode position, and thus that a post-mortem histological verification was not needed for this purpose. Our goal was to investigate the effect of DBS on hippocampal connectivity in general, rather than on the connectivity between hippocampal substructures.

**Fig 3:**
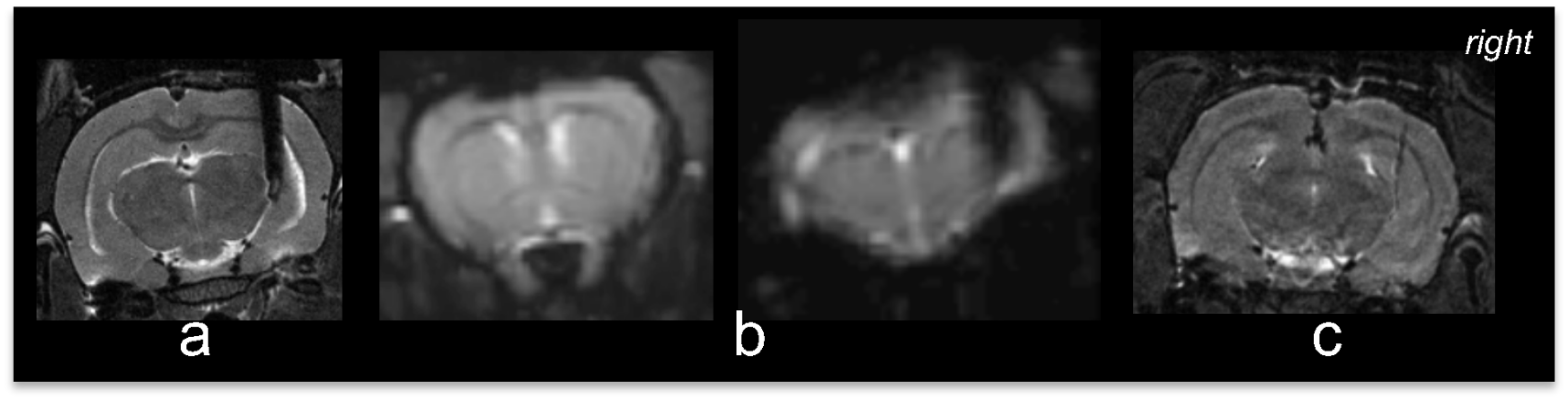
Illustration of electrode-artifact on MR-images. (a) structural MRI of axial slice at height of electrode. (b) functional MRI of axial slice through forebrain. (c) functional MRI of axial slice at height of electrode. (d) post-mortem structural MRI at height of the electrode path. The post-mortem MRI is used to verify the electrode track position. For all rats the electrode track was located in the right hippocampus.

The impact of the electrode artifact on the structural and functional MR image is illustrated in Fig 3a and 3b/3c, respectively. The electrode track obtained via a post-mortem MRI and after careful removal of the electrode, is shown in Fig 3d. We verified that the electrode track was in the right hippocampus for all subjects.

### Data-analysis

Preprocessing was done in SPM8 (http://www.fil.ion.ucl.ac.uk/) on a subject-by-subject basis. First, all images from all sessions were realigned to the first image of the first session, using a least squares approach and a 6 parameter rigid body spatial transformation. Second, all datasets were coregistered to the anatomical image using a normalized mutual information metric. Third, in-plane smoothing was done using a Gaussian kernel with Full Width at Half Maximum (FWHM) of (1x1)mm^2^. Finally, a band pass filter (0.01Hz– 0.08Hz) was applied to reduce low frequency and physiological noise (e.g. breathing (~0.3Hz) and heartbeat (~1.0Hz)). No global signal regression was performed since detrending of the data did not appear to optimize the result. Probably, the filtering step did already suffice to remove low frequency baseline drift (e.g. MR scanner drift and aliasing of physiological pulsations), which is particularly important in brain regions that weakly activate [48]. In order to prepare the preprocessed data for statistical analysis all datasets were normalized on a subject-by-subject basis to their mean, using SPM8.

Statistical data-analysis using the general linear model (GLM), which assumes a known haemodynamic response function (HRF) to a certain stimulus, is the standard way to identify correlated voxels in fMRI data. In this study, we employ a refined data-driven processing technique (Independent Component Analysis, ICA) to analyze our fMRI data. ICA has proven to be useful in extraction of independent components related to brain activations that are difficult to specify beforehand, as well as physiological and non-physiological noise [49]. ICA can extract BOLD responses without imposing constraints on the HRF. Therefore, ICA might detect responses that vary in time and shape, and that could not be detected with GLM analysis. ICA is frequently applied in neuroscience research to estimate functional connectivity of the brain in the resting state [50–51]. This technique divides the BOLD signal into different independent temporally correlated components and allows investigating functional connectivity in the entire brain without the use of prior information [51]. However, limited prior information is included through the selection of the anatomically relevant components by the investigator during interpretation. ICA of the preprocessed fMRI data was performed using GIFT (Group ICA of fMRI toolbox (http://icatb.sourceforge.net/). We used 5 components, except in one subject that required 15 components for a clear separation of the actual activation for the ICA analysis. In this way the components are calculated without splitting up converging regions over different components or compiling non-converging regions into one component [50]. Every activation or response map is an average across 6 fMRI-sessions, acquired at three different scanning days. Only voxels with a Bonferroni corrected p-value below 0.05 were considered active and used in further investigation and visualization. Bonferroni correction was done by dividing the significance level by the number of brain voxels, i.e. post-smoothing. The Bonferroni correction method corrects more strictly than is actually necessary, because it assumes that the data at neighbouring voxels are completely independent from each other. However, in reality, neighbouring voxels show similar activation patterns within functionally defined brain regions. In order to avoid type II errors, we made the correction less strict, by dividing by the number of brain voxels post-smoothing. This number was estimated to be around 10000, since the rat brain consists approximately of 30.000 voxels with dimensions of 0.312x0.312mm^2^ and a smoothing kernel of 1 mm FWHM was used.

The response maps were overlaid on the structural MR. The response maps were not masked. The inter-subject reproducibility was evaluated based on the visual comparison of the mean response map of all subjects.

Prior to ICA analysis, response maps were compiled using standard GLM analysis, by calculating the correlation between the stimulation paradigm and the BOLD response over time for each voxel. A Bonferroni corrected p-value of 0.05 was used as the threshold for display. For the generation of the GLM-based response maps all fMRI sessions were grouped per subject, per stimulation intensity. For completeness, we compared the findings of ICA and GLM analyses.

## Results

Bipolar Poisson distributed hippocampal DBS induces a reproducible positive stimulation intensity-dependent BOLD response in: ipsilateral hippocampal structures (il HC); contralateral hippocampal structures (cl HC); medial thalamic structures (mThal) such as the ventral posteromedial thalamic nucleus (VPM) and mediodorsal thalamic nucleï (MD); lateral thalamic structures (lThal), such as the ventral posterolateral thalamic nucleus (VPL), the reticular thalamic nucleus (Rt), the medial geniculate nucleus (MGN); septal nuclei; striatum; hypothalamus (hypoT); medial forebrain structures (mFB), including the nucleus Accumbens (NAcc); and sensory cortex (SC). In some subjects a positive BOLD response was also observed in other structures, such as the mammillary bodies (MM), the ventral tegmental area (VTA), cingulate cortex (Cg) and ectorhinal cortex (EC). The most reproducible to least reproducible brain structures are listed from top to bottom for every subject in Table 1.

**Table 1:** Inter-subject reproducibility.

Structure	S1	S2	S3	S4	S5	S6	S7
**cl HC**	x	x	x	x	x	x	x
**mThal**	x	x	x	x	-	x	x
**septum**	x	x	x	x	x	x	x
**mFB**	x	x	x	-	x	x	x
**hypoT**	x	x	x	-	x	x	x
**striatum**	x	x	x	-	x	x	x
**il HC**	-	x	x	x	x	x	-
**lThal**	x	-	x	x	-	x	x
**SC**	-	x	x	-	x	x	x
**MM**	-	x	-	-	x	-	-
**VTA**	-	x	-	-	-	-	-
**Cg**	-	-	-	-	x	-	-
**EC**	-	-	-	-	x	-	-

The table shows the brain structures with a significant DBS-induced BOLD response per subject (S1/7). The most reproducible to least reproducible brain structures are listed from top to bottom.

**Fig 4:**
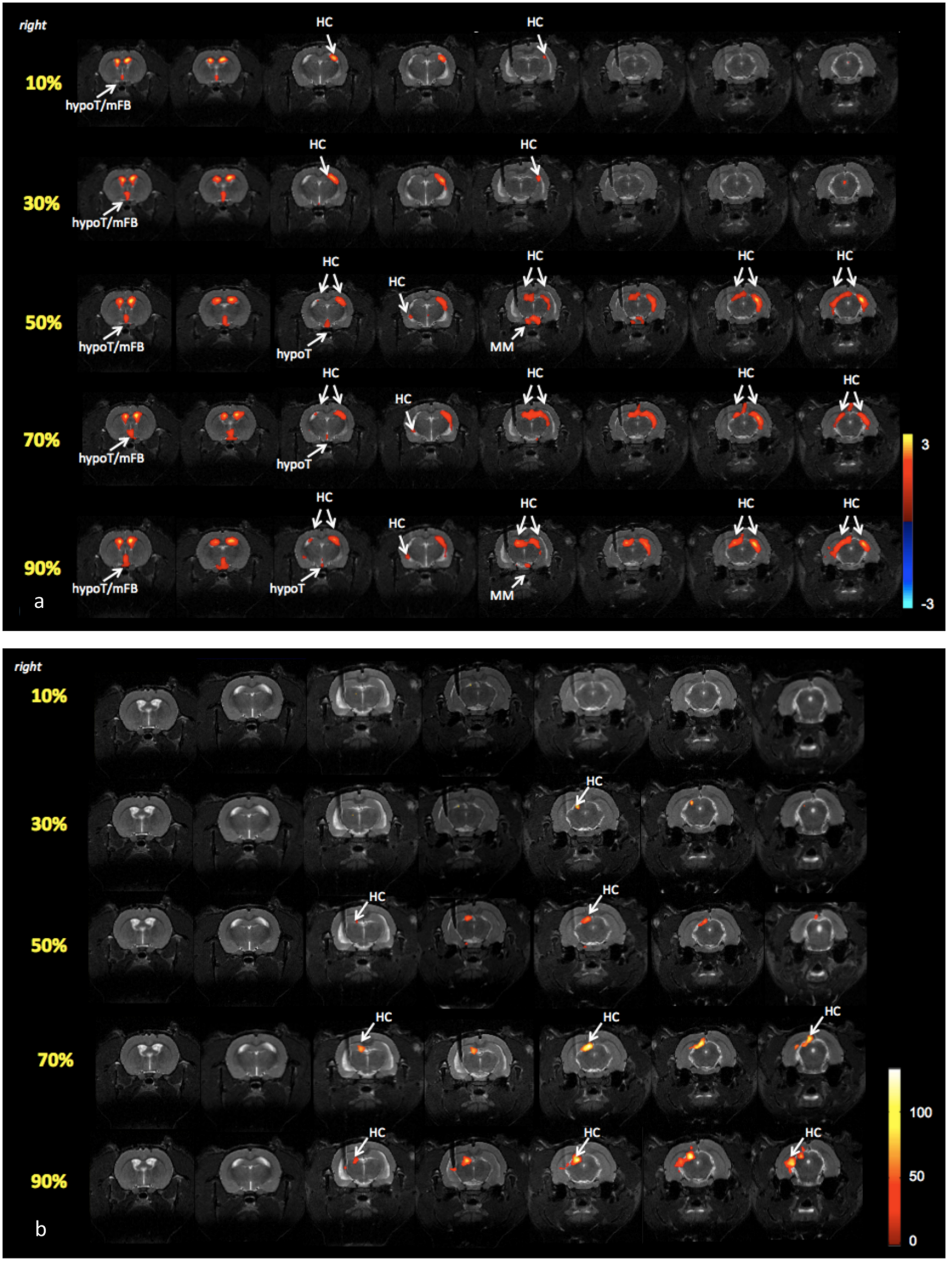
Representative whole-brain BOLD fRMI response-maps of one subject resulting from ICA and GLM analysis. Listed separately in (a) and (b) respectively. All activation maps are thresholded with a Bonferroni corrected p-value < 0.05. Every response map displays the DBS-induced BOLD-response of 5 different stimulation intensities. Every row in the activation map represents a single intensity, listed from top to bottom, from 10% of the seizure threshold to 90% of the seizure threshold. Every row displays the mean of 6 fMRI-datasets for the specific stimulation intensity. Axial anatomical scans are co-registered with the corresponding activation maps. Slices progress from most anterior at the left to most posterior at the right. The hippocampal structures (HC), lateral thalamic structures (lThal), medial thalamic structures (mThal), septal nuclei (septum), striatum, hypothalamus (hypoT), medial forebrain (mFB), sensory cortex (SC), mammillary bodies (MM), ventral tegmental area (VTA), and ectorhinal cortex (EC) are labeled with white arrows. (a) ICA-based response maps. The intensity of the color corresponds to the level of significance of the BOLD response, indicated by a z-score in the color bar on the right. (b) SPM-based response maps. The intensity of the color corresponds to the level of correlation of the voxel’s timecourse to the stimulus, indicated by an F-value in the color bar on the right.

The cl HC is reproducible throughout all subjects. mThal, septum, mFB and hypoT are present in 6/7 subjects and il HC, lThal and striatum in 5/7 subjects. Mean whole-brain ICA- and GLM-based response maps are shown for one subject in Fig 4a and 4b respectively. Supplementary figures are added, showing the ICA-based response maps for the six other subjects.

Furthermore, we can conclude that our results are reproducible within each subject (i.e. in-between sessions), since data-analysis of the grouped fMRI sessions (within one subject) results in a mean response map with significant activation in several brain structures, for all subjects.

The BOLD response became more widespread with a higher stimulation intensity for all subjects. A bilateral BOLD response was observed at 90% of the seizure threshold in all subjects, at 70% of the seizure threshold in 5/7 subjects, at 50% of the seizure threshold in 4/7 subjects, and in two subjects already at 30% of the seizure threshold. At 10% of the seizure threshold only a unilateral response was observed in all subjects. These results indicate the impact of the stimulation intensity to the spatial extent of the DBS-induced BOLD response. In each subject the response became more widespread with increasing stimulation intensity. There were no brain structures with a negative BOLD response (after Bonferroni correction) induced by DBS in any subject, at any stimulation intensity.

The unlabeled clusters in Fig 4a, and in S2–S7 Figs, are considered to be fMRI-related artifacts. We observed an artifact in the lateral ventricles (e.g. the two clusters in the first two axial slices in Fig 4a) and in the aqueduct, the small channel connecting the third and fourth ventricles of the rat brain (e.g. the cluster in the latter axial slice in S5 Fig). These are probably artifacts because their cluster size is independent of the stimulation intensity, whilst it is hypothesized that DBS-related clusters become bigger with increasing stimulation intensity.

For completeness of the study, we compared the results from the standard GLM analysis to those from the ICA analysis. Mean whole-brain ICA- and GLM-based response maps are shown for one subject, for all stimulation intensities, in Fig 4a and 4b respectively. Additional information on the GLM analysis can be found in S1 Fig As to be expected, GLM maps and ICA maps show significant correlation. However, GLM analysis reveals only unilateral BOLD responses in brain structures of the hippocampal formation, whereas ICA analysis also reveals bilateral and secondary BOLD responses in target areas of the hippocampus, more distant from the electrode tip. GLM analysis of the individual sessions shows significant activation in secondary brain structures in some sessions, but the response was not reproducible over sessions or within subject.

**Fig 5:**
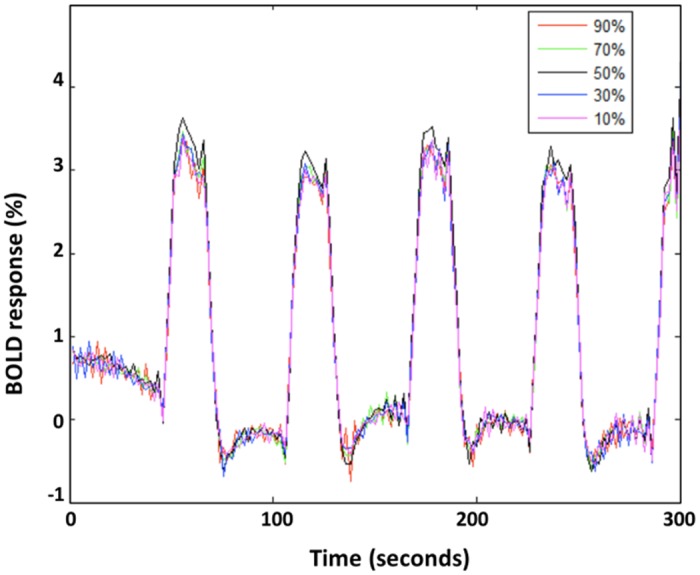
BOLD responses at different stimulation intensities in one subject. The graph depicts the mean timeseries per stimulation intensity in the volume of interest with the highest significance, per stimulation intensity, in one subject. The volume of interest increased with increasing stimulation intensity.

We were not able to correlate the stimulation intensity to the intensity or the significance of the BOLD response. This is illustrated in Fig 5 for one subject. The graph depicts the mean timecourse of the cluster with the highest correlation to the stimulation paradigm per stimulation intensity. This mean timecourse was calculated using GLM. The purpose of this graph is only to illustrate that the degree of correlation to the stimulation paradigm could not be correlated to the stimulation intensity. However, the size of the used clusters to calculate the mean timecourse increased with increasing stimulation intensity.

## Discussion

### Whole-brain response of DBS

With our simultaneous DBS fMRI experiment we demonstrated that hippocampal DBS, with preclinically proven successful stimulation parameters to treat TLE, significantly affects the BOLD signal in mesial forebrain and mesial temporal brain structures. Secondly, we were able to visualize not only global, but also bilateral responses to in vivo stimulation of the hippocampus, in hippocampal and mesolimbic brain structures. Thirdly, we observed an increasing bilateral connectivity with increasing intensity.

It is well-known that mesial forebrain and mesial temporal structures, as well as bilateral brain structures, have strong commissural connections. FMRI studies of the healthy rat brain have shown robust and reproducible autonomous circuits [52–53]. The observed bilateral synchrony of fMRI signals reflects these inter-hemispheric commissural connections, especially in brain regions with strong commissural connections such as the hippocampus [53]. The current study confirms these findings. The mesolimbic pathway is a dopaminergic pathway in the brain, which begins in the VTA and connects to the NAcc. This might explain why we also observed a significant BOLD response in VTA and mFB/NAcc in some subjects. Clinical and experimental studies investigated the role of the major neuromodulatory systems in epilepsy, such as dopamine [54–56], which is well known to regulate seizure activity. A fMRI study in unilateral MTLE patients has shown important decreases of functional connectivity in the ventromesial limbic forebrain regions and in the NAcc [57]. These regions are involved in long-term memory for novel events and reward. The authors also observed reduced connectivity in the hippocampus, amygdala and default mode network [57]. Neuroimaging studies have shown that the hippocampus and amygdala are also part of the mesolimbic network [58–59]. An ictal iEEG study in MTLE patients has suggested that the mFB is strongly affected by mesial temporal activity [60], indicating a preferential seizure spread from mesial temporal lobes to mesial frontal lobes, consequently leading to reduced connectivity in all brain structures affected by seizure propagation. This reduced functional connectivity in the mesial temporal lobe structures may explain cognitive and psychiatric impairments often found in patients with MTLE. The therapeutic potential of DBS for MTLE patients, for seizure reduction purposes has been hypothesized in literature [19,20,24]. Our study shows a DBS-induced increase in the BOLD signal in mesial temporal lobe structures and mesial frontal lobe structures in healthy rats, strengthening that hypothesis. Further research is necessary to investigate potential DBS-induced restoration of MTLE-induced loss of functional connectivity.

In addition, we observed increasing DBS-induced bilateral connectivity with increasing stimulation intensity. This might indicate that non-responding mesial temporal lobe epilepsy (MTLE) patients become responders to DBS when increasing the stimulation intensity, in order to reach more brain structures of the mesolimbic pathway. These findings are concordant with previous rodent fMRI research of direct stimulation of the perforant pathway of the rat hippocampus. These studies also revealed a positive BOLD response in hippocampal structures and target regions of the hippocampus, indicating that stimulation of the hippocampal formation can activate the mesolimbic pathway [29–34]. FMRI research of direct stimulation of the perforant pathway of the rat hippocampus also revealed a linear relationship between the intensity used to stimulate the hippocampal formation and the extension of the induced BOLD response [32]. These studies indicate that long-term potentiation (i.e. the increased synaptic strength due to the repeated stimulation of hippocampal neurons) increases interhemispheric communication and recruitment of limbic and neocortical circuits after changes in synaptic strength within the hippocampus [35], which might explain why we observed increasing DBS-induced bilateral connectivity with increasing stimulation intensity.

### Statistical analysis

Comparison of the results from the GLM analysis to those from the ICA analysis demonstrates that: (1) ICA analysis is able to replicate the results found with the standard GLM analysis; (2) ICA analysis returns additional information.

Since the GLM map indicates voxel regions that vary according to the a priori defined stimulus, a statistically independent source, ICA analysis should indeed include the brain activation found with GLM.

With GLM we only found a unilateral brain response, whereas with ICA we were able to trace a bilateral brain response in structures remote from the stimulated area. With GLM, each voxel is independently evaluated for a statistical relationship between its timeseries and an a priori modeled stimulus response. ICA analysis is not restricted by the use of a specific modeled response, since it looks for functionally connected regions, which are indirectly dependent, or independent of the stimulus. This could explain why ICA analysis returns more information. Since with ICA, areas with a delayed HRF, remote from the stimulated area, can be traced as well. Our findings coincide with previous task-fMRI studies where both data analysis techniques are compared. The authors found the regions of activation identified by ICA overlapped substantially with those identified by SPM and that ICA was able to separate additional structures. ICA is less sensitive to motion artifacts and therefore could provide better results in datasets corrupted with motion artifacts, e.g. due to changes in the level of sedation [61–62].

### Other functional imaging techniques

Other functional imaging studies have been performed in our lab prior to this study, where we investigated the effect of hippocampal DBS in the healthy rat brain with 2-deoxy-2-[^18^F]fluoro-D-glucose (^18^F-FDG)-PET [63] and 99mTc-hexamethylpropyl-eneamineoxime (99mTc-HMPAO)-SPECT [45]. We observed DBS induced hypometabolism and hypoperfusion in the ispi- and contralateral hippocampal formation and other limbic structures, and found that the intensity of the hypometabolic or hypoperfused region remained unaltered for all stimulation intensities, but that the total volume of hypometabolic or hypoperfused tissue became more widespread with higher stimulation intensities, meaning that higher stimulation intensities lead to larger volumes being stimulated. Similar functional imaging studies have also observed a linear relationship between stimulation intensity and spatial extent of the DBS induced changes [64–65]. Another ^18^F-FDG-PET study investigated the neuronal network activity patterns in the healthy rat brain, affected by STN DBS. They also concluded that unilateral DBS affects brain activity ipsi- as well as contralateral to the stimulation site, which implies a bilateral effectiveness [66].

In contradiction to the DBS induced hypometabolism and hypoperfusion, our DBS-fMRI study indicate hippocampal and mesolimbic excitation instead of inhibition. This may be partially attributed to the difference in time course between the imaging modalities used, since fMRI uses a temporal resolution of seconds and ^18^F-FDG-PET and 99mTc-HMPAO-SPECT have a time window of minutes. Taken together, these studies suggest that DBS might lead to initial (order of seconds) activation of the targeted structure and might lead to a decrease in baseline activity of the targeted structure over time (order of minutes). These findings suggest the hippocampus and other mesial and cortical limbic structures up- and downstream are affected by hippocampal DBS. However, it still remains to be elucidated if these structures are only temporarily affected.

### Limitations

Simultaneous DBS fMRI studies come with a few limitations: (1) the electrode artifact affects the quality of the EPI around the electrode, potentially causing underestimation of the BOLD response at the site of the electrode. This might explain why we mainly observed a contralateral hippocampal response as opposed to an ipsilateral hippocampal response. (2) Deviations in electrode placement, smaller than the spatial resolution of the MR images, may lead to large differences in response. This might explain the inter-subject variability of the affected brain structures, as shown in Table 1. (3) DBS might cause temporary changes in cellular activity that might be overlooked with fMRI. (4) The BOLD response to a stimulus involves a complex interaction of the cerebral blood flow (CBF), cerebral blood volume (CBV) and cerebral metabolic rate of oxygen (CMRO_2_) [6]. This complex interaction within the fMRI signal makes it difficult to interpret BOLD fMRI data alone. Additional fMRI studies, such as CBV, CBF, and functional connectivity studies, and cellular studies might provide complementary information. (5) MRI is generally considered to be a safe imaging technique, as opposed to PET and SPECT, which are prone to the risk of exposure to ionizing radiation. However, the presence of intracerebral metal makes MRI a potentially dangerous technique in patients with subcutaneously DBS units. Safety risks include heating, local tissue damage due to high frequency currents and electrode displacement, induced by the MR gradient coils or/and radiofrequent pulses [67–70]. Despite the presence of intracerebral metal, neuronal function is unlikely to be affected by the high frequency of induced currents [71]. Provided a number of technical precautions are taken, MRI can be regarded as safe even in patients implanted with a DBS system [67,72–73]. Carmichael et al. reported that temperature increases sufficiently low to suggest that thermal or electromagnetically mediated experimental confounds to fMRI with DBS are unlikely [74]. Additionally, in patients with a DBS-system implanted, MRI is used routinely to control for correct implantation or more recently, for MR-guided implantation of the electrode [74–76]. However, further studies are required to rule out safety risks of MRI in patients implanted with a DBS system.

Due to an elaborate pre-experimental preparation we were able to establish an experimental protocol (i.e. the surgery, DBS and scan protocol) that minimized these limitations. Because we did not observe any signs of local tissue damage on the post-mortem structural MR images, and the BOLD response to DBS was found to be consistent, we can conclude we established a safe and robust experimental design.

## Conclusion

With this study, we demonstrate that simultaneous DBS fMRI is able to trace distal and bilateral responses to circuit activation on a large spatial scale, and that the brain volume affected by DBS increases with an increasing stimulation intensity. To our knowledge this is the first DBS fMRI study with a chronically implanted MRI-compatible electrode for hippocampal DBS. We showed that our experimental protocol, for longitudinal DBS fMRI studies, provides reproducible results. Our results demonstrate that hDBS robustly modulates the mesolimbic network. This finding may hold clinical relevance for hippocampal DBS therapy in epilepsy cases, as connectivity in this network has previously been shown to be suppressed in mTLE. Further research is necessary to investigate potential DBS-induced restoration of MTLE-induced loss of functional connectivity in mesolimbic brain structures. Clinical simultaneous DBS fMRI studies could be complemented with behavioral and cognitive evaluation in order to achieve the visualization of therapeutic DBS circuits. In this way, translation to clinical investigation of existing as well as new parameters and targets for DBS might lead to the reduction of non-responders to the treatment.

## Supporting Information

S1 ARRIVE Checklist ARRIVE checklist. (PDF)

S1 Fig Representative DBS fMRI result of one subject, obtained with GLM analysis. The response map is the result of 6 sessions grouped together of one subject, at a stimulation intensity of 90% of the threshold. A positive BOLD response can be clearly observed in the ipsilateral hippocampus. The response map is thresholded at p < 0.05, after Bonferroni correction.
(TIF)

S2 Fig Individual whole-brain BOLD fRMI response map of subject 2, resulting from ICA analysis. The response map is thresholded with a Bonferroni corrected p-value < 0.05. The intensity of the color corresponds to the level of significance of the BOLD response, indicated by a z-score in the color bar on the right. The response map displays the DBS-induced BOLD-response of 5 different stimulation intensities. Every row in the response map represents a single intensity, listed from top to bottom, from 10% of the seizure threshold to 90% of the seizure threshold. Every row displays the mean of 6 fMRI-datasets for the specific stimulation intensity. Axial anatomical scans are co-registered with the corresponding activation maps. Slices progress from most anterior at the left to most posterior at the right. The hippocampal structures (HC), medial thalamic structures (mThal), septal nuclei (septum), striatum, hypothalamus (hypoT), medial forebrain (mFB), mammillary bodies (MM) and ventral tegmental area (VTA) are labeled with white arrows.
(TIF)

S3 Fig Individual whole-brain BOLD fRMI response map of subject 3, resulting from ICA analysis. The response map is thresholded with a Bonferroni corrected p-value < 0.05. The intensity of the color corresponds to the level of significance of the BOLD response, indicated by a z-score in the color bar on the right. The response map displays the DBS-induced BOLD-response of 5 different stimulation intensities. Every row in the response map represents a single intensity, listed from top to bottom, from 10% of the seizure threshold to 90% of the seizure threshold. Every row displays the mean of 6 fMRI-datasets for the specific stimulation intensity. Axial anatomical scans are co-registered with the corresponding activation maps. Slices progress from most anterior at the left to most posterior at the right. The hippocampal structures (HC), lateral thalamic structures (lThal), medial thalamic structures (mThal), septal nuclei (septum), striatum, hypothalamus (hypoT), medial forebrain (mFB) and sensory cortex (SC) are labeled with white arrows.
(TIF)

S4 Fig Individual whole-brain BOLD fRMI response map of subject 4, resulting from ICA analysis. The response map is thresholded with a Bonferroni corrected p-value < 0.05. The intensity of the color corresponds to the level of significance of the BOLD response, indicated by a z-score in the color bar on the right. The response map displays the DBS-induced BOLD-response of 5 different stimulation intensities. Every row in the response map represents a single intensity, listed from top to bottom, from 10% of the seizure threshold to 90% of the seizure threshold. Every row displays the mean of 6 fMRI-datasets for the specific stimulation intensity. Axial anatomical scans are co-registered with the corresponding activation maps. Slices progress from most anterior at the left to most posterior at the right. The hippocampal structures (HC), lateral thalamic structures (lThal), medial thalamic structures (mThal), septal nuclei (septum), striatum, hypothalamus (hypoT), medial forebrain (mFB) and sensory cortex (SC) are labeled with white arrows.
(TIF)

S5 Fig Individual whole-brain BOLD fRMI response map of subject 5, resulting from ICA analysis. The response map is thresholded with a Bonferroni corrected p-value < 0.05. The intensity of the color corresponds to the level of significance of the BOLD response, indicated by a z-score in the color bar on the right. The response map displays the DBS-induced BOLD-response of 5 different stimulation intensities. Every row in the response map represents a single intensity, listed from top to bottom, from 10% of the seizure threshold to 90% of the seizure threshold. Every row displays the mean of 6 fMRI-datasets for the specific stimulation intensity. Axial anatomical scans are co-registered with the corresponding activation maps. Slices progress from most anterior at the left to most posterior at the right. The hippocampal structures (HC), lateral thalamic structures (lThal), medial thalamic structures (mThal), septal nuclei (septum), striatum, hypothalamus (hypoT), medial forebrain (mFB), and sensory cortex (SC) are labeled with white arrows.
(TIF)

S6 Fig Individual whole-brain BOLD fRMI response map of subject 6, resulting from ICA analysis. The response map is thresholded with a Bonferroni corrected p-value < 0.05. The intensity of the color corresponds to the level of significance of the BOLD response, indicated by a z-score in the color bar on the right. The response map displays the DBS-induced BOLD-response of 5 different stimulation intensities. Every row in the response map represents a single intensity, listed from top to bottom, from 10% of the seizure threshold to 90% of the seizure threshold. Every row displays the mean of 6 fMRI-datasets for the specific stimulation intensity. Axial anatomical scans are co-registered with the corresponding activation maps. Slices progress from most anterior at the left to most posterior at the right. The hippocampal structures (HC), lateral thalamic structures (lThal), medial thalamic structures (mThal) and septal nuclei (septum) are labeled with white arrows.
(TIF)

S7 Fig Individual whole-brain BOLD fRMI response map of subject 7, resulting from ICA analysis. The response map is thresholded with a Bonferroni corrected p-value < 0.05. The intensity of the color corresponds to the level of significance of the BOLD response, indicated by a z-score in the color bar on the right. The response map displays the DBS-induced BOLD-response of 5 different stimulation intensities. Every row in the response map represents a single intensity, listed from top to bottom, from 10% of the seizure threshold to 90% of the seizure threshold. Every row displays the mean of 6 fMRI-datasets for the specific stimulation intensity. Axial anatomical scans are co-registered with the corresponding activation maps. Slices progress from most anterior at the left to most posterior at the right. The hippocampal structures (HC), lateral thalamic structures (lThal), medial thalamic structures (mThal), septal nuclei (septum), striatum, hypothalamus (hypoT), medial forebrain (mFB) and sensory cortex (SC) are labeled with white arrows.
(TIF)
