# Supplementary figures and images for: Functional MRI during Hippocampal Deep Brain Stimulation in the Healthy Rat Brain
Source: PLoS One. 2015 Jul 20;10(7):e0133245. doi: 10.1371/journal.pone.0133245 (PMC4508110; doi:10.1371/journal.pone.0133245)

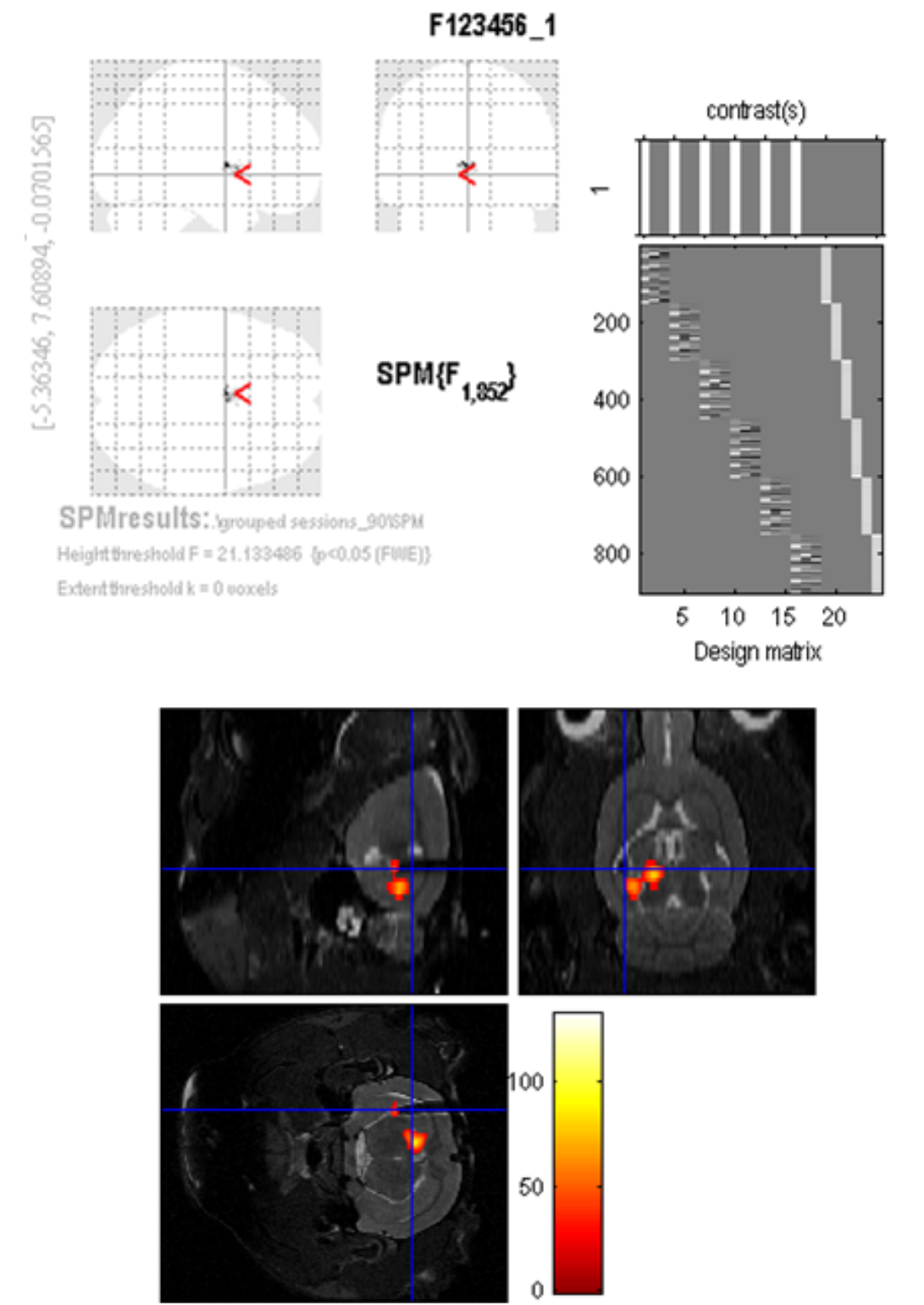

Supplement: S1 Fig — The response map is the result of 6 sessions grouped together of one subject, at a stimulation intensity of 90% of the threshold. A positive BOLD response can be clearly observed in the ipsilateral hippocampus. The response map is thresholded at p < 0.05, after Bonferroni correction. (TIF) [file pone.0133245.s002.tif]

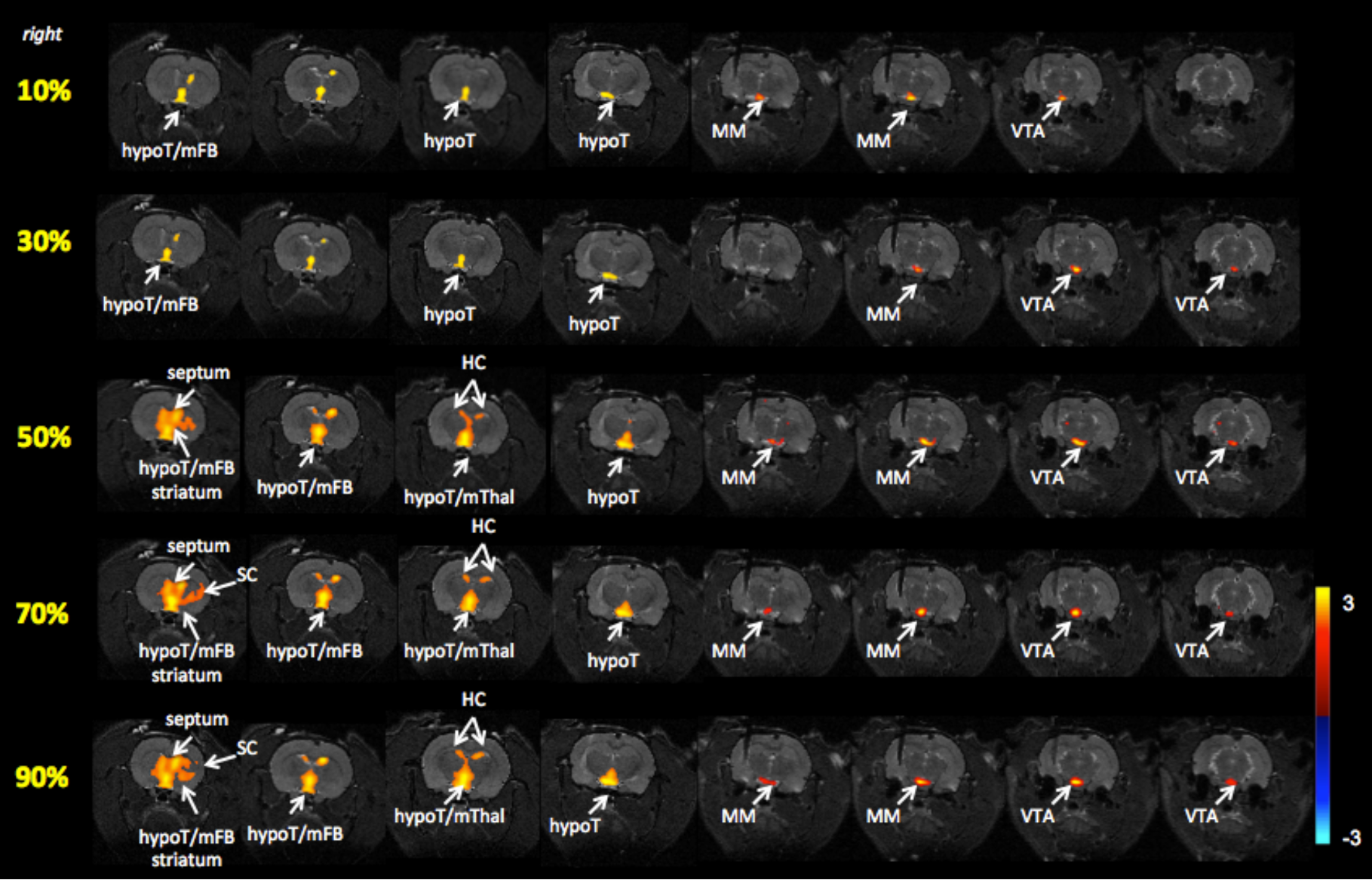

Supplement: S2 Fig — The response map is thresholded with a Bonferroni corrected p-value < 0.05. The intensity of the color corresponds to the level of significance of the BOLD response, indicated by a z-score in the color bar on the right. The response map displays the DBS-induced BOLD-response of 5 different stimulation intensities. Every row in the response map represents a single intensity, listed from top to bottom, from 10% of the seizure threshold to 90% of the seizure threshold. Every row displays the mean of 6 fMRI-datasets for the specific stimulation intensity. Axial anatomical scans are co-registered with the corresponding activation maps. Slices progress from most anterior at the left to most posterior at the right. The hippocampal structures (HC), medial thalamic structures (mThal), septal nuclei (septum), striatum, hypothalamus (hypoT), medial forebrain (mFB), mammillary bodies (MM) and ventral tegmental area (VTA) are labeled with white arrows. (TIF) [file pone.0133245.s003.tif]

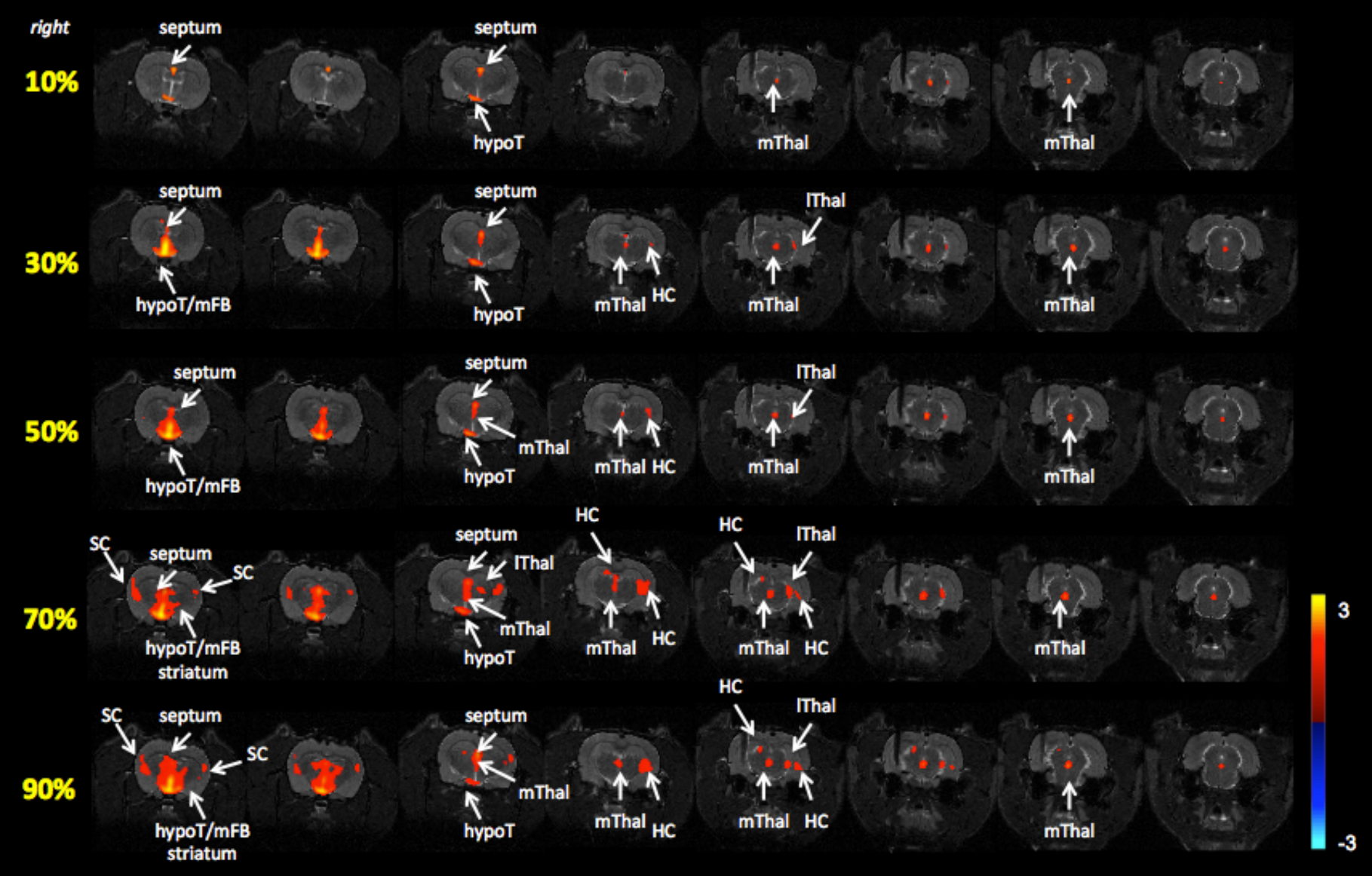

Supplement: S3 Fig — The response map is thresholded with a Bonferroni corrected p-value < 0.05. The intensity of the color corresponds to the level of significance of the BOLD response, indicated by a z-score in the color bar on the right. The response map displays the DBS-induced BOLD-response of 5 different stimulation intensities. Every row in the response map represents a single intensity, listed from top to bottom, from 10% of the seizure threshold to 90% of the seizure threshold. Every row displays the mean of 6 fMRI-datasets for the specific stimulation intensity. Axial anatomical scans are co-registered with the corresponding activation maps. Slices progress from most anterior at the left to most posterior at the right. The hippocampal structures (HC), lateral thalamic structures (lThal), medial thalamic structures (mThal), septal nuclei (septum), striatum, hypothalamus (hypoT), medial forebrain (mFB) and sensory cortex (SC) are labeled with white arrows. (TIF) [file pone.0133245.s004.tif]

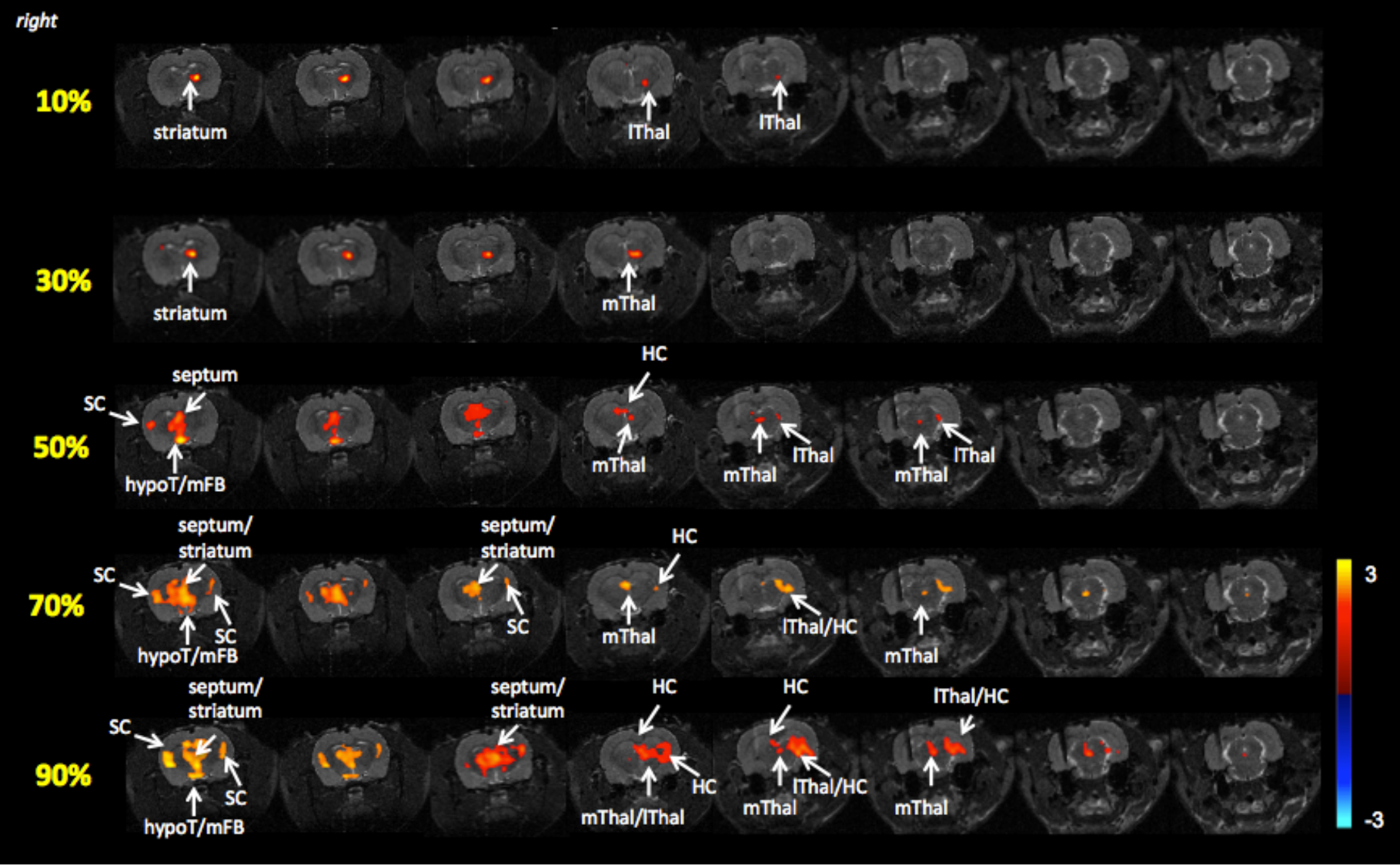

Supplement: S4 Fig — The response map is thresholded with a Bonferroni corrected p-value < 0.05. The intensity of the color corresponds to the level of significance of the BOLD response, indicated by a z-score in the color bar on the right. The response map displays the DBS-induced BOLD-response of 5 different stimulation intensities. Every row in the response map represents a single intensity, listed from top to bottom, from 10% of the seizure threshold to 90% of the seizure threshold. Every row displays the mean of 6 fMRI-datasets for the specific stimulation intensity. Axial anatomical scans are co-registered with the corresponding activation maps. Slices progress from most anterior at the left to most posterior at the right. The hippocampal structures (HC), lateral thalamic structures (lThal), medial thalamic structures (mThal), septal nuclei (septum), striatum, hypothalamus (hypoT), medial forebrain (mFB) and sensory cortex (SC) are labeled with white arrows. (TIF) [file pone.0133245.s005.tif]

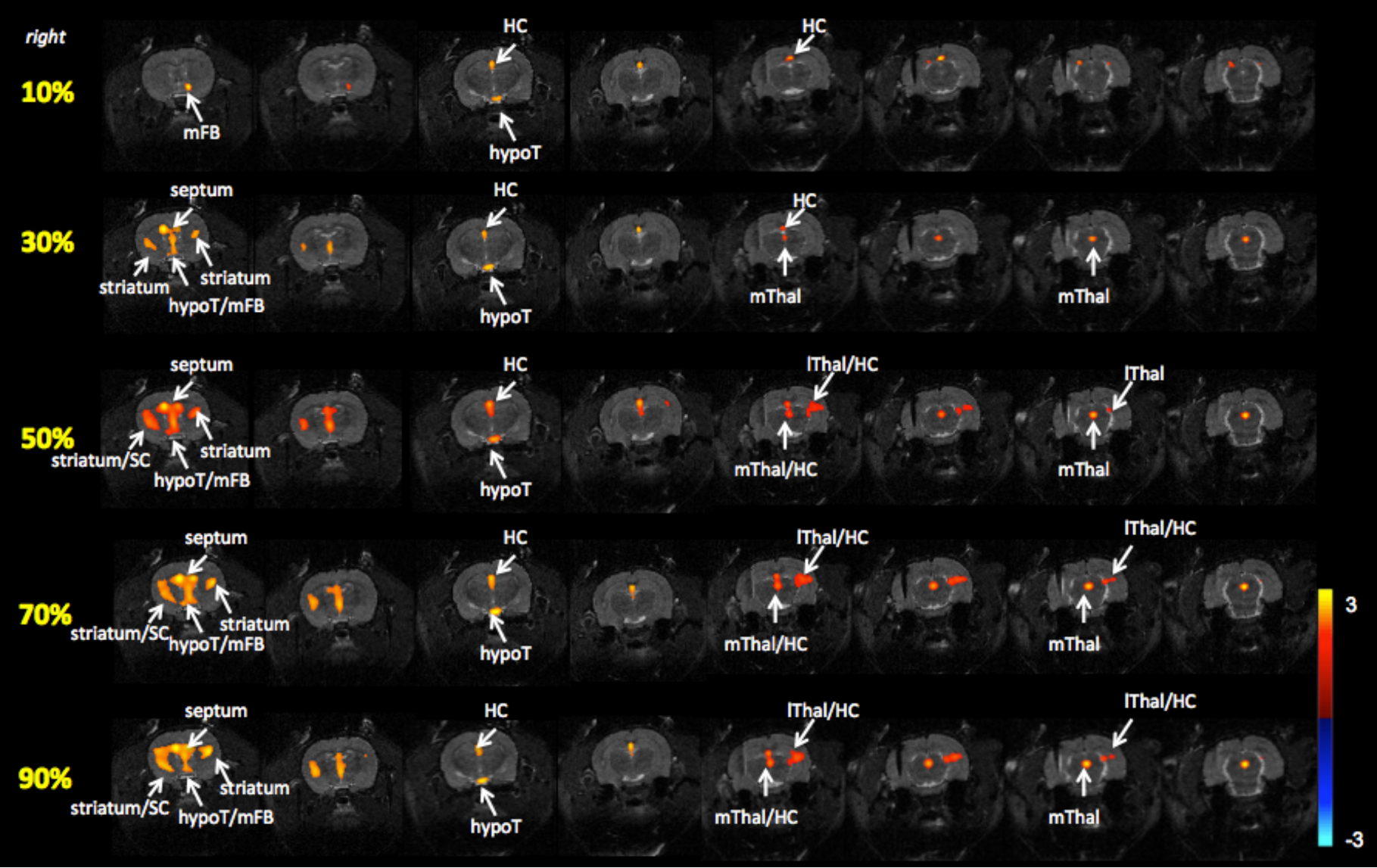

Supplement: S5 Fig — The response map is thresholded with a Bonferroni corrected p-value < 0.05. The intensity of the color corresponds to the level of significance of the BOLD response, indicated by a z-score in the color bar on the right. The response map displays the DBS-induced BOLD-response of 5 different stimulation intensities. Every row in the response map represents a single intensity, listed from top to bottom, from 10% of the seizure threshold to 90% of the seizure threshold. Every row displays the mean of 6 fMRI-datasets for the specific stimulation intensity. Axial anatomical scans are co-registered with the corresponding activation maps. Slices progress from most anterior at the left to most posterior at the right. The hippocampal structures (HC), lateral thalamic structures (lThal), medial thalamic structures (mThal), septal nuclei (septum), striatum, hypothalamus (hypoT), medial forebrain (mFB), and sensory cortex (SC) are labeled with white arrows. (TIF) [file pone.0133245.s006.tif]

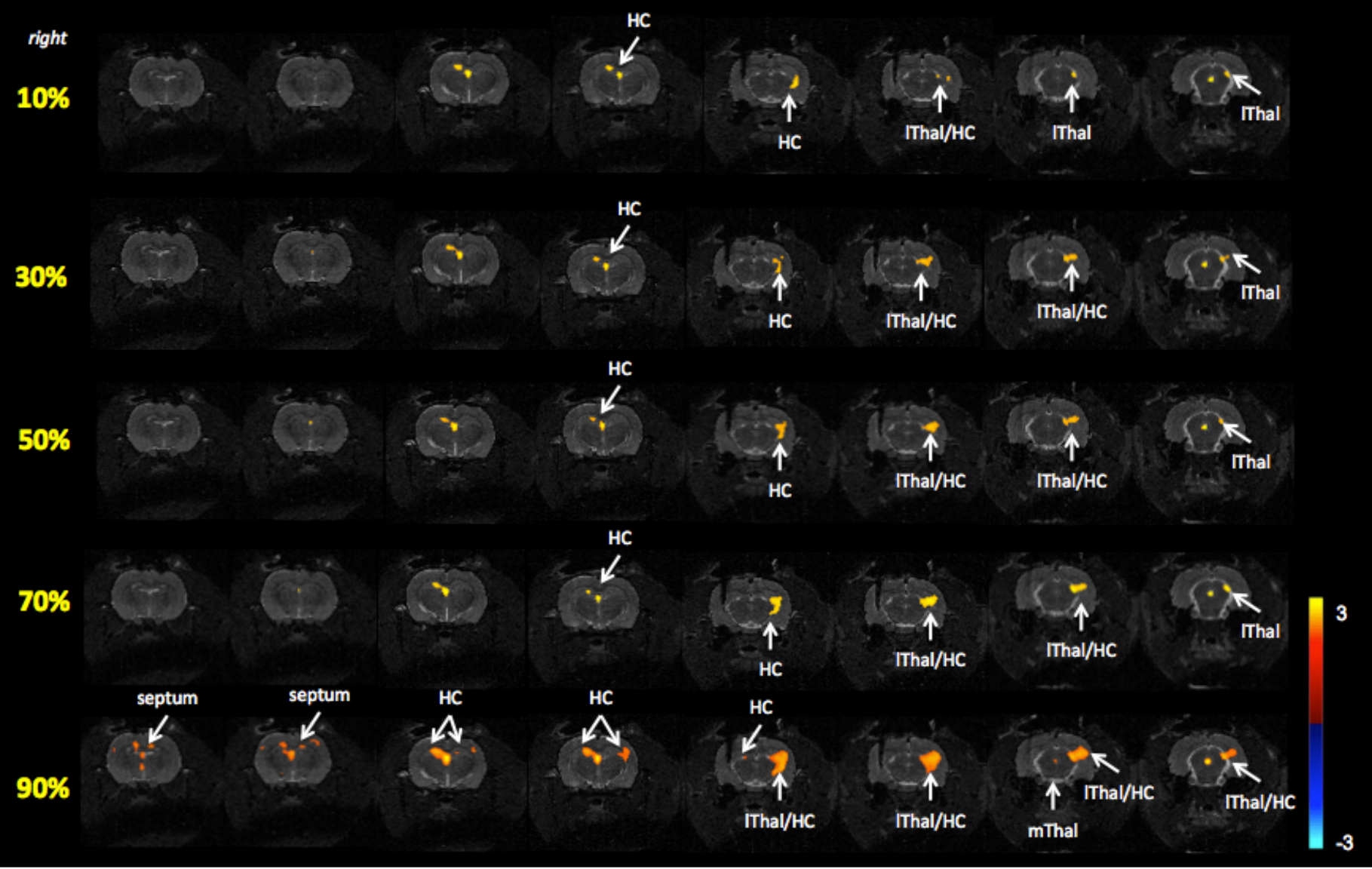

Supplement: S6 Fig — The response map is thresholded with a Bonferroni corrected p-value < 0.05. The intensity of the color corresponds to the level of significance of the BOLD response, indicated by a z-score in the color bar on the right. The response map displays the DBS-induced BOLD-response of 5 different stimulation intensities. Every row in the response map represents a single intensity, listed from top to bottom, from 10% of the seizure threshold to 90% of the seizure threshold. Every row displays the mean of 6 fMRI-datasets for the specific stimulation intensity. Axial anatomical scans are co-registered with the corresponding activation maps. Slices progress from most anterior at the left to most posterior at the right. The hippocampal structures (HC), lateral thalamic structures (lThal), medial thalamic structures (mThal) and septal nuclei (septum) are labeled with white arrows. (TIF) [file pone.0133245.s007.tif]

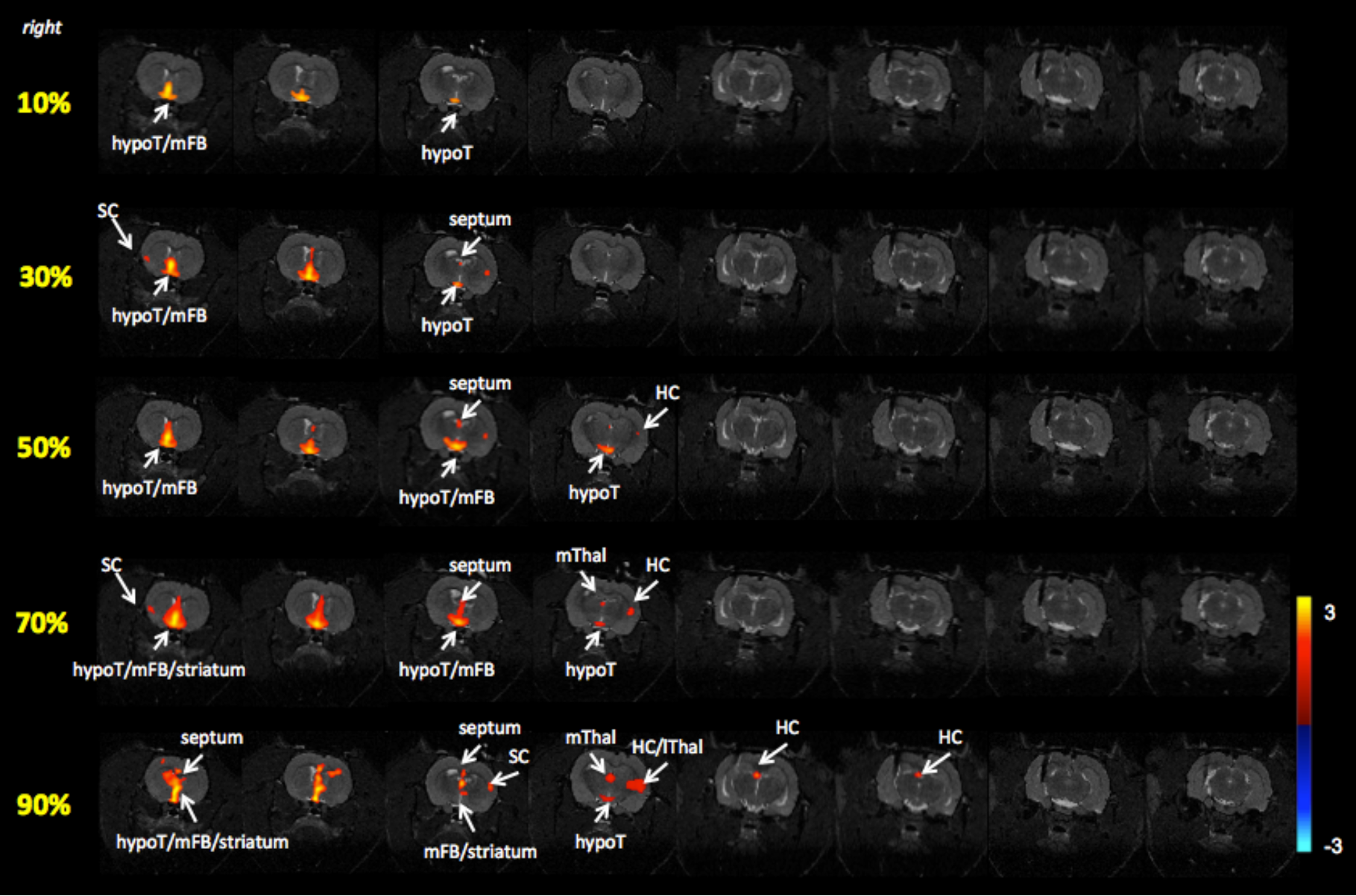

Supplement: S7 Fig — The response map is thresholded with a Bonferroni corrected p-value < 0.05. The intensity of the color corresponds to the level of significance of the BOLD response, indicated by a z-score in the color bar on the right. The response map displays the DBS-induced BOLD-response of 5 different stimulation intensities. Every row in the response map represents a single intensity, listed from top to bottom, from 10% of the seizure threshold to 90% of the seizure threshold. Every row displays the mean of 6 fMRI-datasets for the specific stimulation intensity. Axial anatomical scans are co-registered with the corresponding activation maps. Slices progress from most anterior at the left to most posterior at the right. The hippocampal structures (HC), lateral thalamic structures (lThal), medial thalamic structures (mThal), septal nuclei (septum), striatum, hypothalamus (hypoT), medial forebrain (mFB) and sensory cortex (SC) are labeled with white arrows. (TIF) [file pone.0133245.s008.tif]
